# Supplementary material for: Long-Term Synaptic Plasticity Tunes the Gain of Information Channels through the Cerebellum Granular Layer
Source: Biomedicines. 2022 Dec 8;10(12):3185. doi: 10.3390/biomedicines10123185 (PMC9775043; doi:10.3390/biomedicines10123185)
Supplement: Supplementary file 1 [file biomedicines-10-03185-s001.zip › biomedicines-2016696-supplementary.pdf]

Supplementary Materials

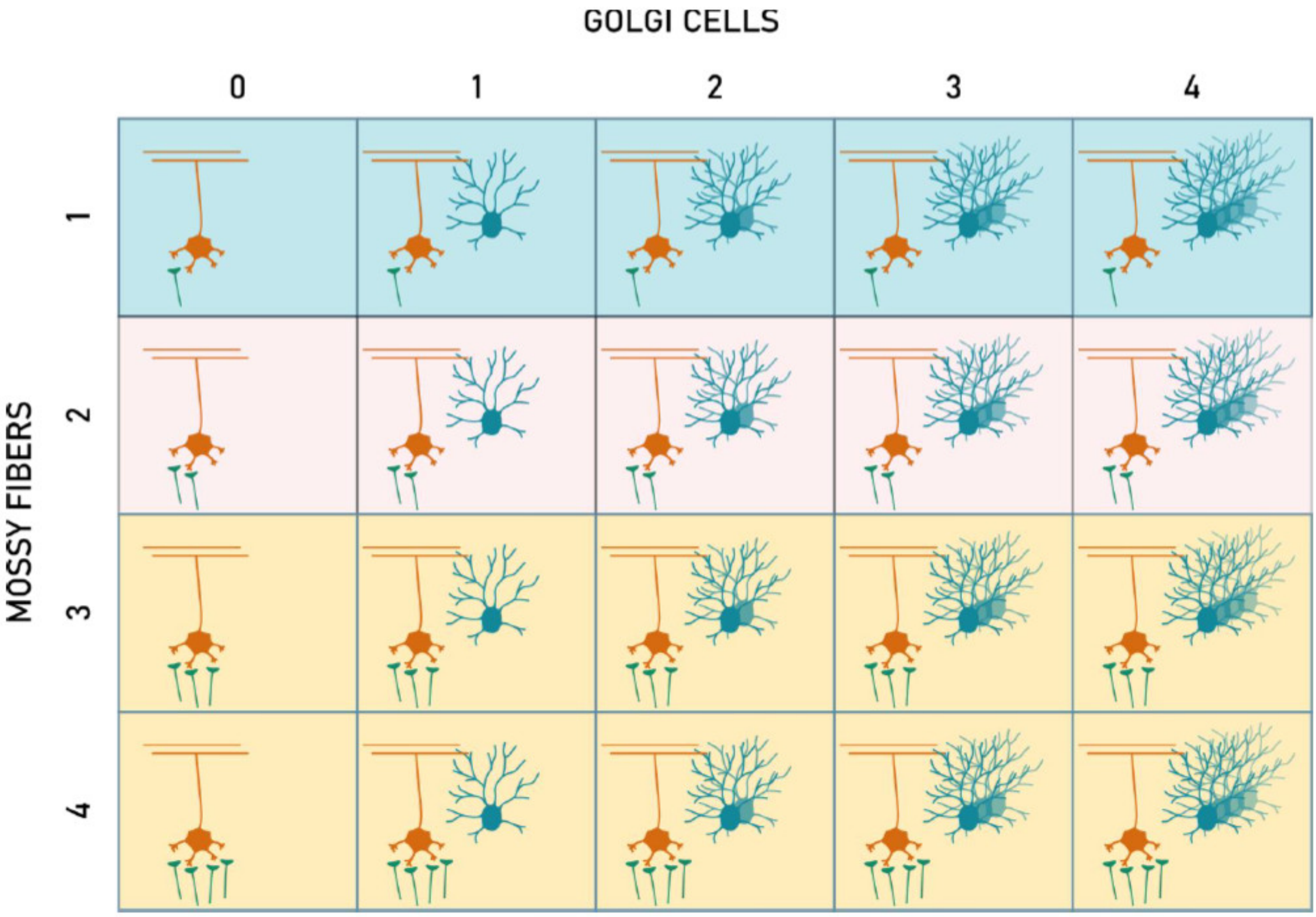

Figure S1. Scheme of the granular layer microcircuit. Panels show the different combinations of mfs and GoCs cells activated during GrC activity simulation.

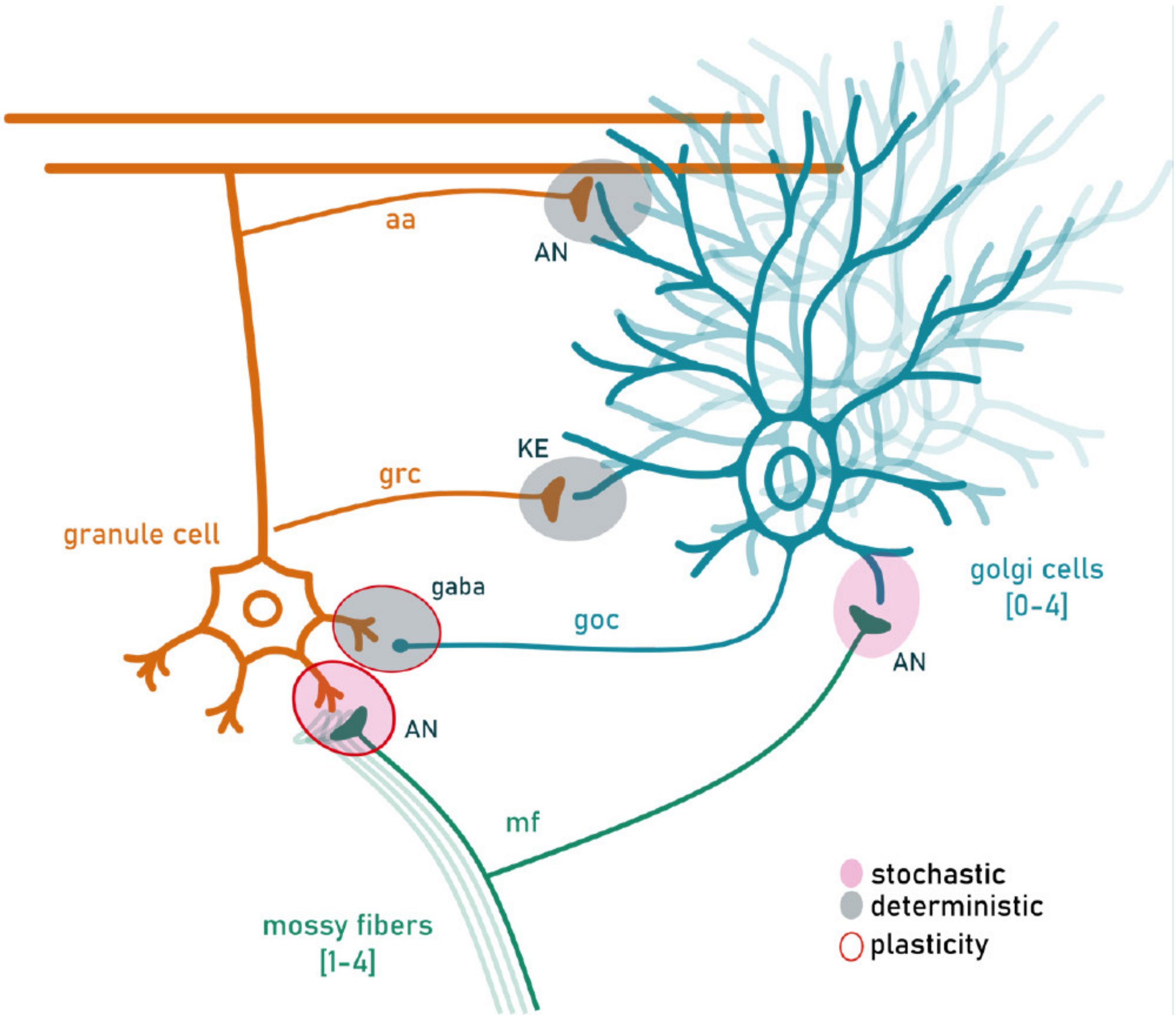

Figure S2. Scheme of the granular layer microcircuit. The stimulating electrode (stim) is positioned onto the mossy fiber bundle (mf) in order to activate excitatory synapses. GoC, Golgi cell (local interneuron); GrC, granule cell (output cell). Glutamatergic AMPA and NMDA synapses (AN); Kainate synapses (KE).

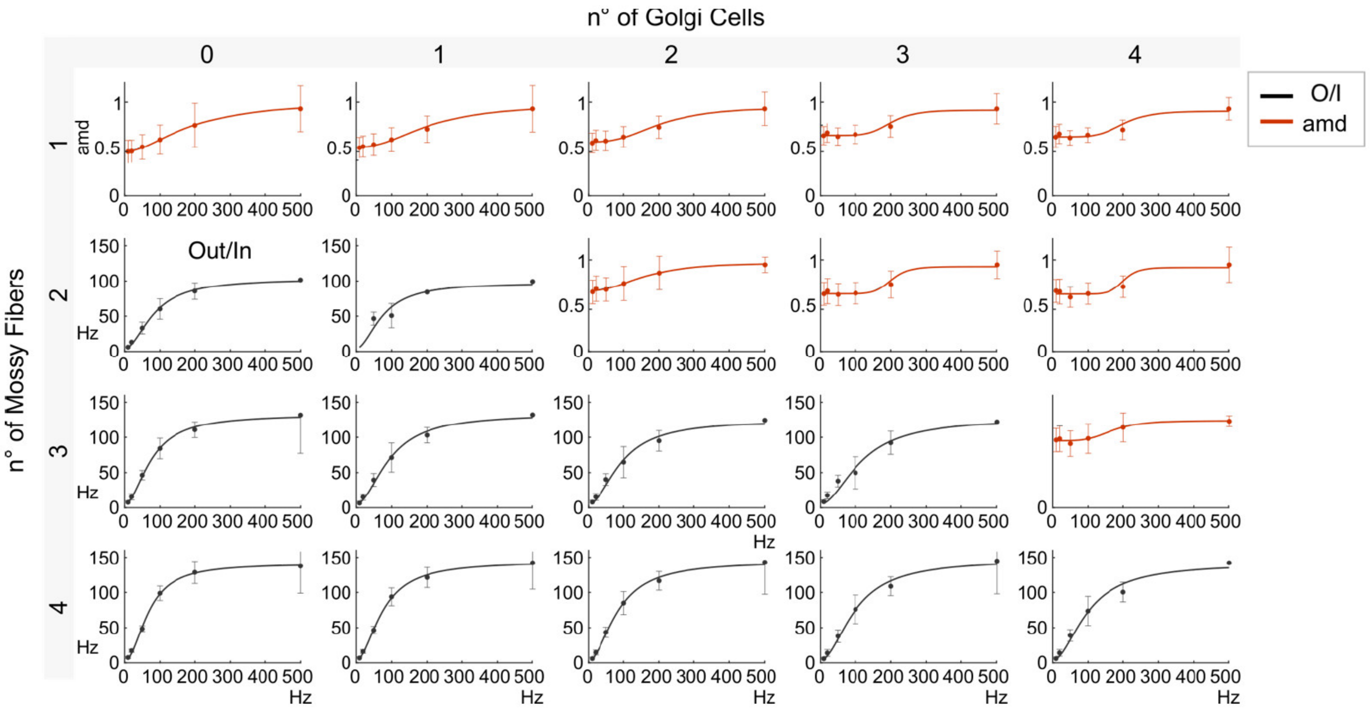

Figure S3. Input/Output responses for all the tested configurations with increasing mossy fibers on the rows (from top to 24 down) while increasing Golgi cells are on the columns (from left to right). Red traces represent cells responding with subthreshold activity and the parameter plotted is *amd*. Black traces represent cells showing spiking responses and the parameter plotted is the output firing frequency. Note the increasing total gain in cells with increasing E/I (lower rows) while higher cutoff frequencies are displayed by cells in columns on the right (lower E/I).
